# Modeling Reef Fish Biomass, Recovery Potential, and Management Priorities in the Western Indian Ocean

**DOI:** 10.1371/journal.pone.0154585

**Published:** 2016-05-05

**Authors:** Timothy R. McClanahan, Joseph M. Maina, Nicholas A. J. Graham, Kendall R. Jones

**Affiliations:** 1 Wildlife Conservation Society, Marine Programs, Coral Reef Conservation Project, Mombasa, Kenya; 2 Australian Research Council Centre of Excellence for Environment Decisions, Centre for Biodiversity and Conservation Science, School of Biological Sciences, The University of Queensland, St Lucia, Queensland 4072, Australia; 3 Department of Environmental Sciences, Macquarie University, North Ryde, New South Wales 2109, Australia; 4 Australian Research Council Centre of Excellence for Coral Reef Studies, James Cook University, Townsville, Queensland 4811, Australia; 5 Lancaster Environment Centre, Lancaster University, Lancaster, LA1 4YQ, United Kingdom; 6 School of Geography, Planning and Environmental Management, University of Queensland, St Lucia, Queensland 4072, Australia; Università di Genova, ITALY

## Abstract

Fish biomass is a primary driver of coral reef ecosystem services and has high sensitivity to human disturbances, particularly fishing. Estimates of fish biomass, their spatial distribution, and recovery potential are important for evaluating reef status and crucial for setting management targets. Here we modeled fish biomass estimates across all reefs of the western Indian Ocean using key variables that predicted the empirical data collected from 337 sites. These variables were used to create biomass and recovery time maps to prioritize spatially explicit conservation actions. The resultant fish biomass map showed high variability ranging from ~15 to 2900 kg/ha, primarily driven by human populations, distance to markets, and fisheries management restrictions. Lastly, we assembled data based on the age of fisheries closures and showed that biomass takes ~ 25 years to recover to typical equilibrium values of ~1200 kg/ha. The recovery times to biomass levels for sustainable fishing yields, maximum diversity, and ecosystem stability or conservation targets once fishing is suspended was modeled to estimate temporal costs of restrictions. The mean time to recovery for the whole region to the conservation target was 8.1(± 3SD) years, while recovery to sustainable fishing thresholds was between 0.5 and 4 years, but with high spatial variation. Recovery prioritization scenario models included one where local governance prioritized recovery of degraded reefs and two that prioritized minimizing recovery time, where countries either operated independently or collaborated. The regional collaboration scenario selected remote areas for conservation with uneven national responsibilities and spatial coverage, which could undermine collaboration. There is the potential to achieve sustainable fisheries within a decade by promoting these pathways according to their social-ecological suitability.

## Introduction

Achieving sustainability in fisheries is often challenging due to a lack of data and unclear goals or targets for management [1]. This is particularly true for poor and developing countries [2–4]. The challenge of sustainable fishing has been accentuated by the emergent drive for more holistic ecosystem-based management goals that propose broader ecological and social outcomes, including setting fisheries targets above potential ecological thresholds [4]. One way to handle this complexity is to find proxy metrics that cause or are closely associated with ecological change and can be directly affected, and potentially managed, by human usage. Fish biomass has been shown to be a key proxy for coral reefs where the state of reef ecosystems and the life history composition of the fish community are well predicted by a simple biomass metric [5–8].

Coral reefs in the western Indian Ocean (WIO), the Caribbean and globally have been shown to follow a predictable decline in ecosystem state, processes and potential services as fish biomass diminishes under heavy fishing [5–8]. For the Indian Ocean, this gradient ranges from 7500 kg/ha for the large seascape wilderness of the Chagos Islands [9], to 1200 kg/ha in national coastal fisheries closures [10], to <600 kg/ha in various fisheries [5, 11]. Along a biomass gradient there are changes in ecological processes of carnivory and herbivory, the organic and inorganic carbonate balance, and numbers of species, their life histories, and ecological functions [5, 11, 12].

The first measurable ecological changes appear to emerge when biomass is below ~1050 kg/ha, but changes in number of species and fish life histories occur in succession below 600 kg/ha [11, 12], and degradation of ecological states, processes, and services below 300 kg/ha [5]. Maximum sustained yields have been estimated to occur between 300 and 600 kg/ha, where sustainability includes maintenance of stocks, ecological states, and moderate diversity [5]. Conservation targets, where measured ecological processes are maintained in fished seascapes, are estimated at ~2 standard deviations above the mean estimate of the switch-point for the first measured ecological change, which is 1150 kg/ha [6].

Three targets for planning fisheries are therefore the mid-range estimate for sustainable production (~450 kg/ha), the point where fish diversity declines (~600 kg/ha), and where reef states and processes begin to change (~1150 kg/ha). With these targets and knowledge of the fish biomass or benefits and recovery times or costs, models can optimize the selection of reefs for fisheries restrictions. Previous studies have shown that human population density and particularly distance to markets are good predictors of fish biomass and functional groups [13–15]. Recovery rates are also being increasingly understood from studies of well-enforced long-term fisheries closures [7, 16, 17]. Similar patterns of recovery are emerging in disparate locations, with rate and duration depending on the initial biomass and rates of increase for various functional groups [7]. This emerging information makes it possible to map the distribution of reef fish biomass using proxies for fishing pressure, and to predict recovery rates based on local demography and management conditions. Recovery time can then be evaluated as a cost—the lost opportunity to capture fish–and can be minimized to develop regional fisheries and conservation prioritization plans.

In this study, we modeled the factors that influence fish biomass, and estimated recovery rates under alternative management scenarios in the western Indian Ocean. We present a regional case study where 20% of the reefs are targeted for conservation and 50% for sustainable fishing, which aligns with a call by the Convention for Biological Diversity to put 20% of near-shore areas in closures by 2020 [18]. We then consider three priority-area selection scenarios, two that reduce the costs (recovery time) for two governance scenarios where WIO countries plan and minimize costs independently and collaboratively [19]. Thirdly, where the priority is to raise biomass to multi-species maximum sustained yield (MMSY) levels in degraded reefs. The last scenario is typical of community led closures where local communities prioritize overfished reefs for closure and recovery [20, 21]. The first two scenarios are more typical of national and regional strategies that propose to reach national and global protected area targets [22, 23]. By considering these different assumptions about management needs and governance capacity, we provide a basis for considering a number of likely priority actions.

## Materials and Methods

Our analyses and subsequent mapping utilized 541 surveys of the biomass of fish in 337 sites in the WIO during the period of 1987–2014 by two people (T.R. McClanahan and N.A.J. Graham). All diurnally active, non-cryptic reef associated fish species were included in the surveys. Replicate belt transects were used in some countries, and replicate stationary point counts in others, which have been shown to yield similar biomass values in methodological comparisons [24, 25]. Species or family level fish survey data at a site over time were pooled into total fish biomass values (kg/ha) for each site. Sites were grouped into five different fisheries management categories as follows: remote sites (isolated reefs far from human populations); high compliance closure (133 sites); low compliance and young closure (73 sites); all destructive gear restricted (43 sites, only line and trap fishing permitted); most destructive gear restricted (154 sites, spear guns and gill nets also used); and no gears restricted (95 sites, small mesh seine nets and explosives also in use) (S1 Fig). Sites on the isolated Indian Ocean atolls were categorized as ‘remote’ (54 sites). These groups were further categorized into either fished or un-fished. These groupings were based on maps of protected areas and the authors experience working in the above study sites [6]. For each record, the time period (in years) during which the corresponding management type was implemented was also recorded. Site attributes, including the Euclidean distance to the nearest town (i.e. potential fish markets) and the population of the town was added for each record. We defined a market as a national capital, provincial capital, major population center, or landmark city, following Cinner et al. [26, 27]. Population data associated with each market records were extracted from the Gridded Population of the World (GPW) database (CIESIN, 1996; http://sedac.ciesin.columbia.edu/plue/cenguide.html, retrieved Dec 15, 2013) [28]. Sea surface temperature time series weekly data (SST) for 1980–2014 was extracted from CORTAD database (http://www.nodc.noaa.gov/sog/cortad/) and summarized into minimum, mean and maximum [29].

From the above high compliance closure fish biomass data, we determined the relationship between the duration of protection and fish biomass to estimate recovery times in the region. For the 111 sites in 16 high compliance closures, we plotted age since closure against biomass and recovery time was estimated. The relationship between fish biomass and duration of protection was determined by fitting a three parameter self-starting logistic and asymptote models using the *nls* package in R version 3.2.4 (R Core Team 2014), which optimizes given functions to fit available data [30]. The package has an initial attribute that creates the starting estimates for the parameters in the models, representing asymptote biomass value at the inflection point of the curve and a scale parameter in the biomass axis that estimates the time to recovery equation.

### Ethics statement

Permission for fieldwork was granted from the following agencies: 1. Kenya: National Council of Science and Technology; 2. Mozambique: Eduardo Mondlane University; 3. Mayotte: Head of Equipment, Agriculture and Homing Department; 4. Mauritius: Mauritius Oceanography Institute; 5. Madagascar: Ministère de L¹Environnement et des Forêts, Direction du Système des Aires Protégées; 6. South Africa: Departments of Science and Technology, the Environmental Affairs and Tourism, Ezemvelo Kwa Zulu Natal Wildlife, and the iSimangaiso Wetlands Park Authority; 7. Seychelles: Seychelles Bureau of Standards and Nature Seychelles; 8. Tanzania: Institute of Marine Science, University of Dar-es-salaam; 9. In the Maldives, some of the work was with the Banyan Tree Resort who had a permit to conduct research, and some work was under a permit from the Ministry of Fisheries and Agriculture; 10. The British Indian Ocean Territory: the British Indian Ocean Territory Administration; 11. No permit was required for Comoros but we worked with the Coordinator of the Coral Reef Task Force and Focal point of the Nairobi Convention; 12. No permit was required for Reunion.Field studies did not involve manipulation of any endangered or protected species.

### Fish biomass model

To determine the predictors of fish biomass, a full-generalized additive mixed model (GAMM) was constructed with seven predictor variables and interactions, including: management and fishing (fixed terms), distance to markets and market population (log transformed, spline smoothed terms, k = 5); and average SST, minimum SST, and maximum SST (spline smoothed terms, k = 5). The year of sampling was added as a random intercept in the GAMM models. GAMs are the similar to generalized linear models (GLM’s) in that they relate a response variable to one or multiple independent variables but they also have the property of exploring non-linearity in the relationships using smoothers with no *a priori* assumption on the shape of the relationship. All the statistical methods were assessed using a hierarchical modeling framework to account for fish surveys in multiple years.

Predictors were selected on the basis of prior studies in the scientific literature and ongoing work in this field [26]. We would have liked to include predictors such as coral cover, current speeds, and water quality, however we did not have data for these variables across all of our sites and our scale of resolution. Further, coral cover is strongly influenced by factors such as SST and human influences, which are included in our model. Additional social drivers such as levels of local economic development [31], and geomorphological (connectivity and habitat) could have been included, however we did not have empirical data at a our resolution or spatial scale and some simple proxies used in earlier modeling efforts had poor predictive strength. The purpose was not to have a comprehensive assessment of reef fish biomass predictors, but rather to approximate biomass with well-known predictors with moderate to high predictive ability.

This set of predictors was used to construct all possible sub-models, including an intercept-only model, using the dredge function implemented in the MuMIn package [32] (R Core Team 2014). A number of models in the set differed in their data fit by only small amounts, as defined by Akaike Information Criteria (AICc). We therefore employed a model averaging approach to 95% confidence set, a procedure that accounts for model selection uncertainty to obtain robust parameter estimates or predictions [33]. This procedure entails calculating a weighted average of parameter estimates (using Akaike weights), such that parameter estimates from models that contribute little information about the variance in the response variable are given little weight [34], while ameliorating the effects of uninformative parameters [35].

### Spatial prediction of biomass and time to recovery

Using the WCMC coral reef distribution data as the coral reef habitats template (http://www.unep-wcmc.org/), we created 2.5km x 2.5km square grids of ‘planning units’ in the WIO seascape. While there are no standard rules on determining the appropriate planning unit grid size, there are some factors that are useful guidelines, including range size of the species being modeled, area typically utilized by resource users, and the research questions being asked. In consideration of these, we chose a 2.5km grid. This captures the range size of most reef fishes, is representative of the relatively local nature of most reef fishing in the region, and is appropriate for the scale of our region wide study area (~10000 sq km). For each planning unit, site attributes used in fish biomass modeling above were added (i.e. fisheries and closure management categorization, distance to market and population of the market). Using the averaged biomass model constructed above, biomass was predicted spatially on all the planning units before applying the logistic and asymptote model parameters for calculating the time to recovery for each grid; that is the time it takes for fish biomass to recover to a given level of fish biomass. Although social-ecological conditions may change in the future influencing these recovery models, our current data span large gradients in human use, including protected areas embedded in heavily fished seascapes. We calculated time to recovery to three biomass thresholds as possible management targets as described above.

### Priority-area selection to minimize time to recovery

After predicting time to recovery for both sustainable fishing and conservation targets, we evaluated different spatial prioritization approaches, one focused on achieving sustainable fishing and conservation targets at the lowest costs for national and regional scales and the other focused on allowing fish biomass to recover in the most biomass-depleted reefs. The first approach is a complementarity-based spatial prioritization, aimed at identifying sites for protection that complement, rather than replicate, each other. The second approach is a threshold-based spatial prioritization, aimed at selecting all sites that meet pre-established thresholds. Both these approaches are widely used for identifying important areas for biodiversity[36].

We used a spatial prioritization tool, Marxan with Zones [37] to prioritize for marine management areas that minimize the time to recovery. Marxan with Zones uses a simulated annealing algorithm to identify sites that fulfill pre-determined quantitative targets for biodiversity features while minimizing cost, and also allows for the selection of zones with different management actions. In this study, our biodiversity feature is fishable biomass, which represents diversity but also other ecological services [5, 38]. Our costs are the time for fish biomass to recover to the proposed biomass thresholds, which is an opportunity cost of lost catch. By using these times to recovery values as “costs” Marxan minimized time to recovery while meeting the biomass targets. Marxan was given the aim to reserve 20% and 50% of the total reef area as conservation and sustainable fishing zones.

The effect that cross-country collaboration would have on spatial prioritization outcomes for a coordinated international and independent national analysis was examined [22, 39]. In the uncoordinated *Marxan* analysis, the 20% conservation and 50% sustainable fishing targets were met separately for planning units in the Exclusive Economic Zone of each country, while in the coordinated analysis these targets were met across all planning units. For each scenario we conducted 100 *Marxan* runs, and we present these results by identifying 20% of planning units with the highest selection frequency as conservation zones, then removing these planning units and identifying 50% of planning units with the highest selection frequency as sustainable fishing zones. The remaining planning units were classified as unmanaged. The three simple groups were mapped to view management priorities because, given the large scale of the analysis and the relatively small planning units used, it was difficult to view priorities for the entire region using standard *Marxan* selection frequency maps.

The final prioritization approach is entirely threshold-based and was focused on recovering fish biomass in the most biomass-depleted reefs, which is a method that is more focused on recovering sustainability using closures rather than achieving conservation targets. Here, we spatially executed analyses that set and ranked the most depleted planning units (i.e. <450kg/ha) as the priority for closure by reclassifying the grids along with the planning units within a 2.5km radius. Consequently, the planning units selected in the most depleted planning units were designated as ‘core priority areas’ and the adjacent areas as ‘spillover areas’. We then calculated the total areal coverage for both core and spillover areas and estimated the time to recovery of core areas to the above sustainability and conservation targets and mapped these data and present summaries for each location or country.

## Results

### Fish biomass predictions

**Fig 1:**
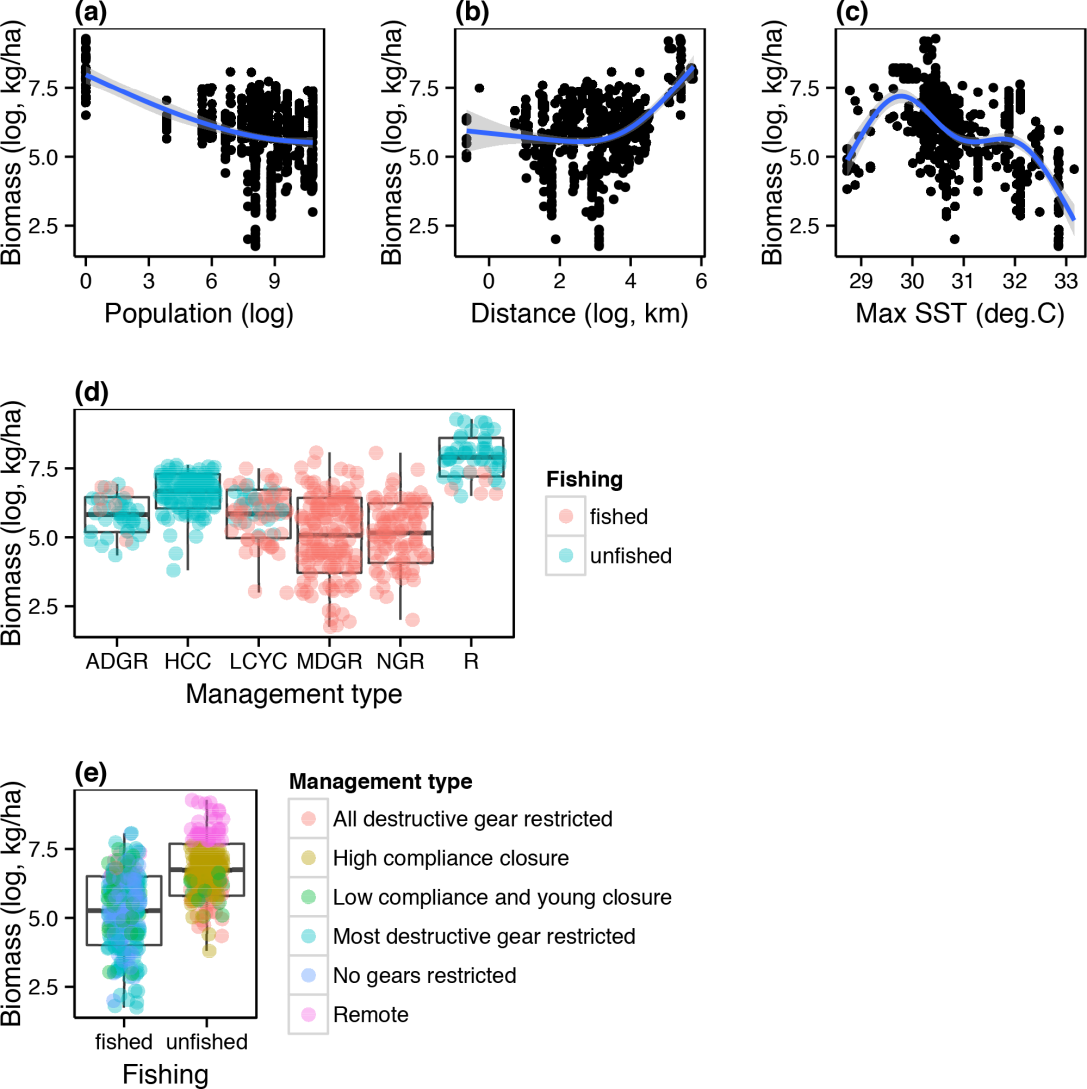
Scatterplots showing the empirical relationships between fish biomass, fisheries management categories, and proxies for the impacts of fishing (i.e. population and distance to markets). These relationships are based on 214 2.5 x 2.5 km cells where fish biomass data were collected and used to develop a regional biomass model for the total of 11678 2.5 x 2.5 km cells in the region with coral reefs (see Fig 3). ADGR = all destructive gear restricted, HCC = high compliance closure, LCYC = low compliance and young closure, MDGR = most destructive gear restricted, NGR = no gear restricted, R = remote.

Among the fish biomass models, the most parsimonious model (Akaike weight = 0.46) explained up to 66% (adjusted R^2^) of the variability observed in fish biomass data (Table 1, Fig 1). Fisheries management type was one of the three most important predictors with high compliance closure and remote management categories having a positive influence on fish biomass, and no gear and most destructive gear restrictions a negative influence (Table 1, Fig 1). Similarly, distance to market and its interaction with reefs that were fished significantly influenced fish biomass, with biomass increasing with increase in distance interaction with ‘fished’ fishing category. Biomass was also influenced by mean maximum SST with had a hump-shaped response pattern, where biomass increased with the maximum SST up to 30°C but declined above those levels. The 95% confidence set of models comprised of four models (Table 1; S1 Table). The second ranked model (Akaike weight = 0.39; R^2^ = 66%) included the fished category in addition to variables in the best model had essentially the same values of the maximized log-likelihood and within 2 AIC as the best model, indicating that the fished category was a non-informative parameter in this model. The third and fourth ranked models (Akaike weights = 0.07, 0.05; R^2^ = 66) comprised all the variables in the first two models, and market population, which did not have a significant influence on biomass.

**Table 1:** Significance tables for parametric model terms (fixed effects) and smooth terms for the top biomass predictive model.

	Variable	Estimate	SE	t	Pr(>|t|)
a) AICc = 1358	Fixed terms				
R^2^ = 0.66	Intercept	5.5	0.2	33.6	<0.01
	Management: High compliance closure	1.0	0.2	6.7	<0.01
	Management: Low compliance and young closure	0.2	0.1	1	NS
	Management: Most destructive gear restricted	-0.4	0.2	-1.3	0.05
	Management: No gears restricted	-0.4	0.2	-2.3	0.05
	Management: Remote	1.9	0.4	7.2	<0.01
	**Smoothed terms, k = 5**	**Edf**		**F**	**P**
	s(log of distance): Fishing-Fished	3.1		21.7	<0.01
	s(log of distance): Fishing-Unfished	1		0.25	NS
	Sea surface temperature—maximum	3.9		34.3	<0.01

**Fig 2:**
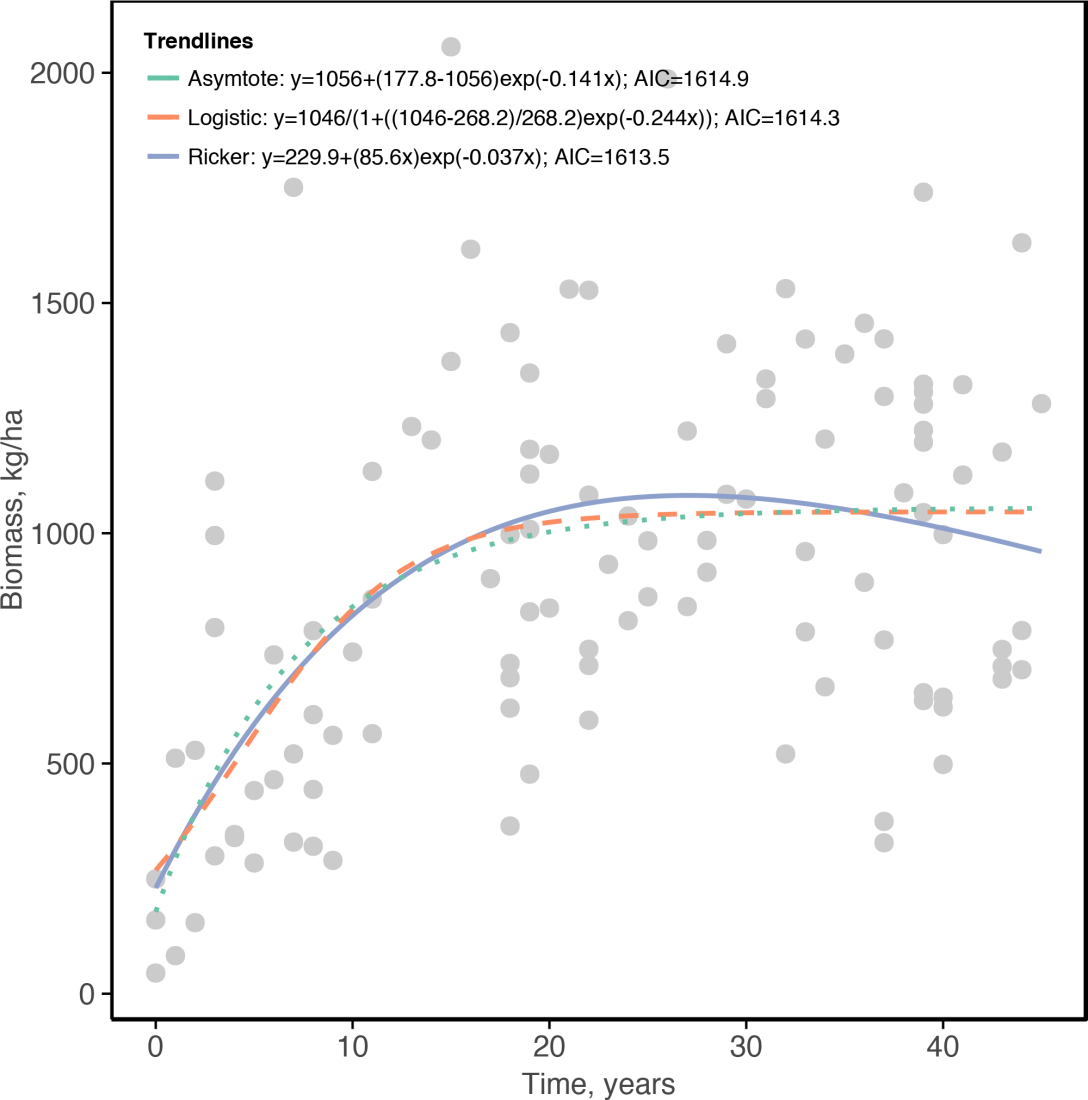
Scatterplot and estimates and best-fit equations for three likely models for the relationship between the age of the high compliance closures and the fish biomass in sampled western Indian Ocean coral reefs.

**Fig 3:**
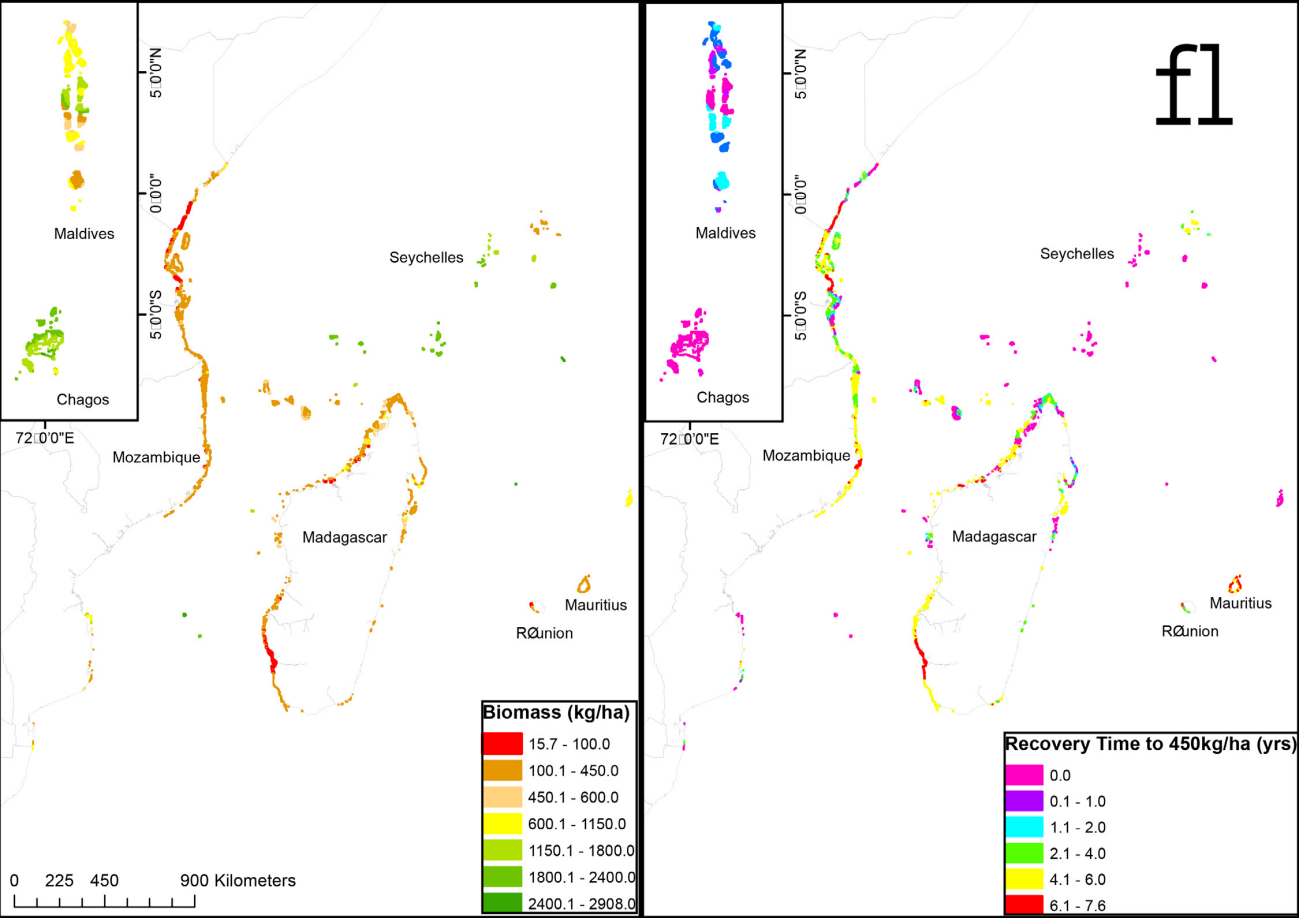
Map of the western Indian Ocean for (a) modeled biomass based on the empirical relationship established in Fig 1, and (b) the estimated time to recover biomass to a mean estimated sustainability level (450 kg/ha).

Most reefs in the region have a fish biomass of less than 600 kg/ha. For example, 42% of the reefs’ cells were predicted to host fish biomass of less than 450kg/ha; 6% more than 450 but less than 600kg/ha; 13% more than 600 and less than 1150; and 39% more than 1150kg/ha (Table 2; Figs 2 and 3A). Notably, the low biomass areas are Kenya’s south and Madagascar’s southwest fringing reefs; with Tanzania, Mozambique, northern Madagascar and most inhabited islands, such as the Comoros, having moderate levels. Maldives is predicted to have highest biomass levels along with remote islands of the Chagos and the Seychelles.

**Table 2:** Modeled biomass (kg/ha) expressed as a percentage of the total reef area for each location or country and the entire western Indian Ocean region.

Country/Biomass	<300 kg/ha	300–450 kg/ha	450–600 kg/ha	600–1150 kg/ha	>1150 kg/ha
Bassas da India	0.00	0.00	0.00	0.00	100.00
British Indian Ocean Territory	0.00	0.00	0.00	3.88	96.12
Comoro Islands	66.03	14.76	19.21	0.00	0.00
Glorioso Islands	72.64	0.00	0.00	0.00	27.36
Ile Europa	0.00	0.00	0.00	0.00	100.00
Ile Tromelin	0.00	0.00	0.00	0.00	100.00
Juan de Nova Island	0.00	0.00	0.00	0.00	100.00
Kenya	32.22	38.57	15.26	13.37	0.58
Madagascar	63.90	21.57	8.66	5.86	0.00
Maldives	0.47	16.22	15.84	42.82	24.66
Mauritius	48.90	0.00	0.00	41.05	10.05
Mayotte	1.81	50.66	42.78	3.53	1.21
Mozambique	80.73	17.07	0.18	1.78	0.23
Reunion	47.77	38.72	0.00	13.51	0.00
Seychelles	3.63	0.54	0.00	0.00	95.83
South Africa	0.00	0.00	0.00	100.00	0.00
Tanzania	44.67	52.93	2.36	0.04	0.00
**Entire Region**	27.22	14.77	6.13	13.28	38.59

### Time to recovery models

The recovery of fish biomass in the high compliance closures indicates good fits to the asymptotic, logistic, and Ricker functions with less than 2 AIC points between the models (Fig 2). Similar response behavior patterns were observed with no significant difference among the three models as indicated by the AIC delta of <2 (Fig 2; S2 Table). Further, all three functions significantly simulated the behavior pattern of the observed data (p<0.01). The data and equations suggest leveling just after 20 years of closure and the average of the logistic and asymptote model parameters were therefore used in the calculations of the time to recovery for the planning units below.

### Mapping recovery times to biomass targets

**Fig 4:**
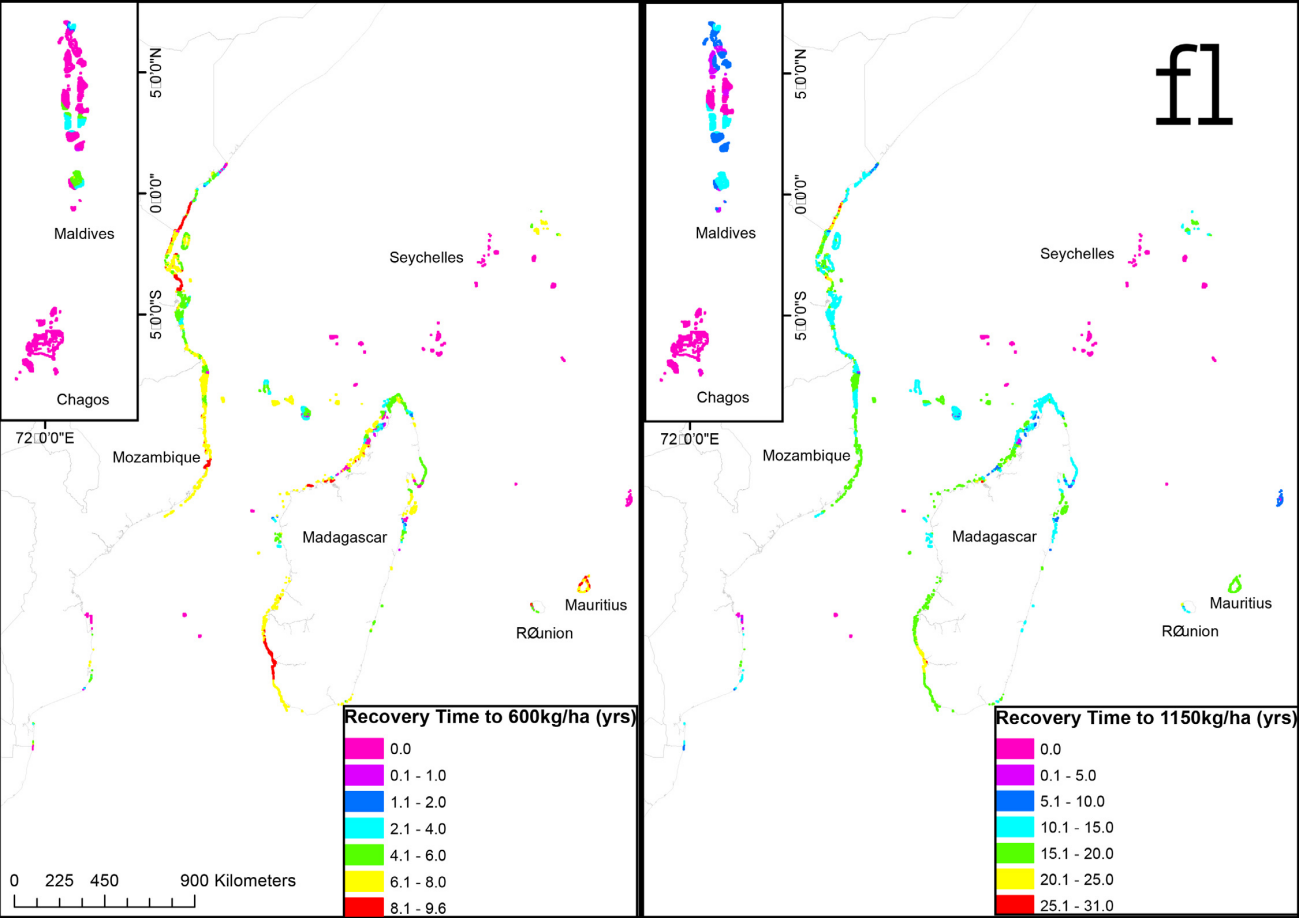
Map of (a) the estimated time to recover biomass to a mean estimated sustainability level (600 kg/ha), and (b) the estimated conservation target of 1150 kg/ha in fully protected fisheries closures studied over a 20-year period (McClanahan et al. 2007).

Three time-to-recovery maps are presented (Figs 3 and 4) based on the projected time to reach the proposed threshold of 1150 kg/ha (Fig 4A and 4B) and the proposed mean production and high diversity sustainability levels of 450 and 600 kg/ha (Figs 3B and 4A). The mean recovery time to the conservation levels for reefs in the region is 8.11±3.02 (± SD) (Table 3). This varies considerably with the initial biomass levels with Kenya’s southwest Madagascar fringing reefs and portions of Mauritius and Reunion requiring over 20–30 years; whereas Tanzania, Mozambique, northern Madagascar and most inhabited islands requiring 5 to 20 years. The remote islands of the Chagos, the outer islands of Seychelles and parts of the Maldives are already above the suggested conservation biomass threshold.

**Table 3:** Mean (±SD) recovery time in years to the three proposed biomass thresholds for each country and the entire Western Indian Ocean region.

	Sustainable Fishing	Maximum Diversity	Conservation
Country	450 kg/ha (SD)	600 kg/ha (SD)	1150 kg/ha (SD)
Bassas da India	0 (0)	0 (0)	0 (0)
British Indian Ocean Territory	0 (0)	0 (0)	0.02 (0.17)
Comoro Islands	3.13 (2.35)	5.72 (1.72)	14.21 (2.48)
Glorioso Islands	3.25 (2.29)	4.62 (3.26)	10.5 (7.4)
Ile Europa	0 (0)	0 (0)	0 (0)
Ile Tromelin	0 (0)	0 (0)	0 (0)
Juan de Nova Island	0 (0)	0 (0)	0 (0)
Kenya	3.11 (2.93)	5.28 (3.04)	15.1 (6.17)
Madagascar	3.43 (2.41)	5.6 (2.47)	14.4 (4)
Maldives	0.13 (0.54)	1.05 (1.77)	6 (4.57)
Mauritius	3.34 (2.96)	4.42 (3.91)	12.36 (6.85)
Mayotte	0.61 (1.06)	3.56 (1.49)	11.05 (2.39)
Mozambique	4.56 (1.51)	6.57 (1.71)	15.3 (3.46)
Reunion	3.59 (2.24)	5.68 (2.59)	14.76 (4.93)
Seychelles	0.71 (1.65)	1.07 (2.43)	2.47 (5.6)
South Africa	0 (0)	0 (0)	6.9 (0.53)
Tanzania	3.65 (1.95)	6.14 (1.41)	14.83 (2.79)
**Entire Region**	1.74 (1.29)	2.92 (1.52)	8.11 (3.02)

The mean recovery time to sustainable yields and maximum diversity levels for reefs in the region are 1.74±1.3 and 2.9±1.5 years, respectively (Table 3). For sustainable yields thresholds, the low initial biomass reefs, southern Kenya’s and southwest Madagascar and portions of Mauritius and Reunion have average recovery periods of 4 to 8 years but the averages for these countries are between 1 to 4 years. In northern Madagascar and most inhabited islands most reefs are already at the two sustainability levels. Tanzania and Mozambique coastlines require variable times ranging from 0 to 7 years. The time to recover maximum diversity showed similar patterns with some time increased to 9 years in the most biomass depleted reefs. Most countries would require 1 to 7 years to reach the maximum diversity threshold at the national level, although some small nations have already achieved this level.

**Fig 5:**
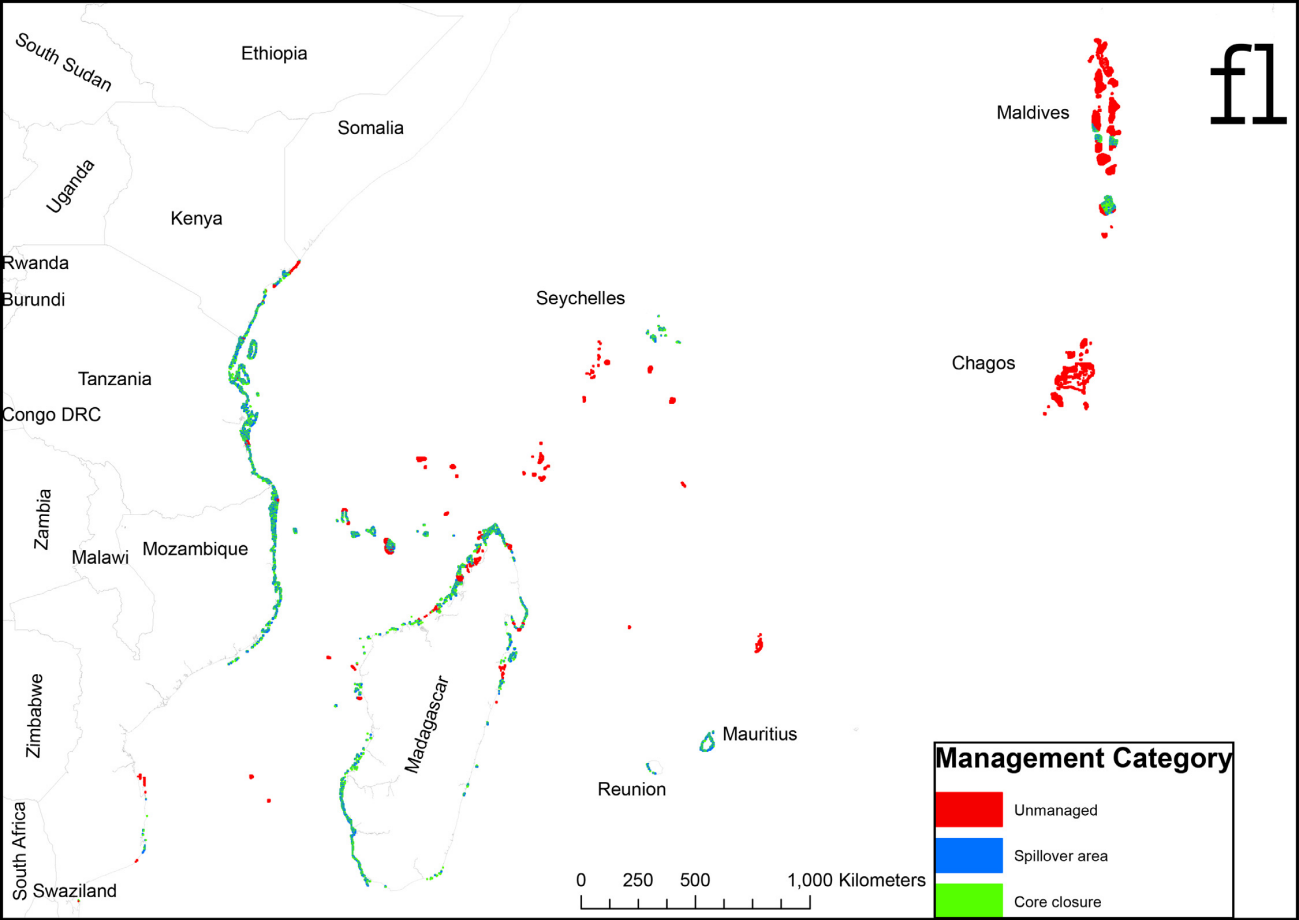
Map derived from algorithm identifying and prioritizing the most depleted fish biomass for small closures and adjacent spillover reefs until all reefs with biomass <450 kg/ha are classified.

Prioritizing placement of closures in the most biomass-depleted reefs and calculating the core priority and adjacent spillover areas indicates that 24.5% of the reef area would be core and 32.6% spillover areas to achieve the threshold of 450 kg/ha for the entire region at our planning unit spatial resolution (Fig 5; Table 4). This also varies considerably between countries with Madagascar, Comoros, and Mauritius requiring ~20–37%, and Reunion, Tanzania, and Mozambique requiring ~40% of their reefs in core areas. The remote offshore islands and Mayotte requiring none to ~30% in this form of management and Kenya with a value of 31% for a highly populated country attributable to a mix of existing national parks and remote areas in northern Kenya with high biomass.

**Table 4:** Amount of reef (as a percentage of the total in each country) selected in each management type using the biomass depletion prioritization scenario.

Country	Core closure (%)	Spillover (%)	Biomass >450
Bassas da India	0.00	0.00	100.00
British Indian Ocean Territory	0.00	0.00	100.00
Comoro Islands	37.53	43.45	19.01
Glorioso Islands	29.46	43.18	27.36
Ile Europa	0.00	0.00	100.00
Ile Tromelin	0.00	0.00	100.00
Juan de Nova Island	0.00	0.00	100.00
Kenya	30.72	47.48	21.80
Madagascar	37.43	49.36	13.20
Maldives	7.65	9.86	82.49
Mauritius	20.88	28.02	51.10
Mayotte	23.77	35.47	40.77
Mozambique	41.74	56.07	2.18
Reunion	40.09	59.91	0.00
Seychelles	1.65	2.52	95.83
South Africa	0.00	0.00	100.00
Tanzania	42.78	55.57	1.64
**Regional Average**	24.49	32.63	42.88

### Spatial planning

**Fig 6:**
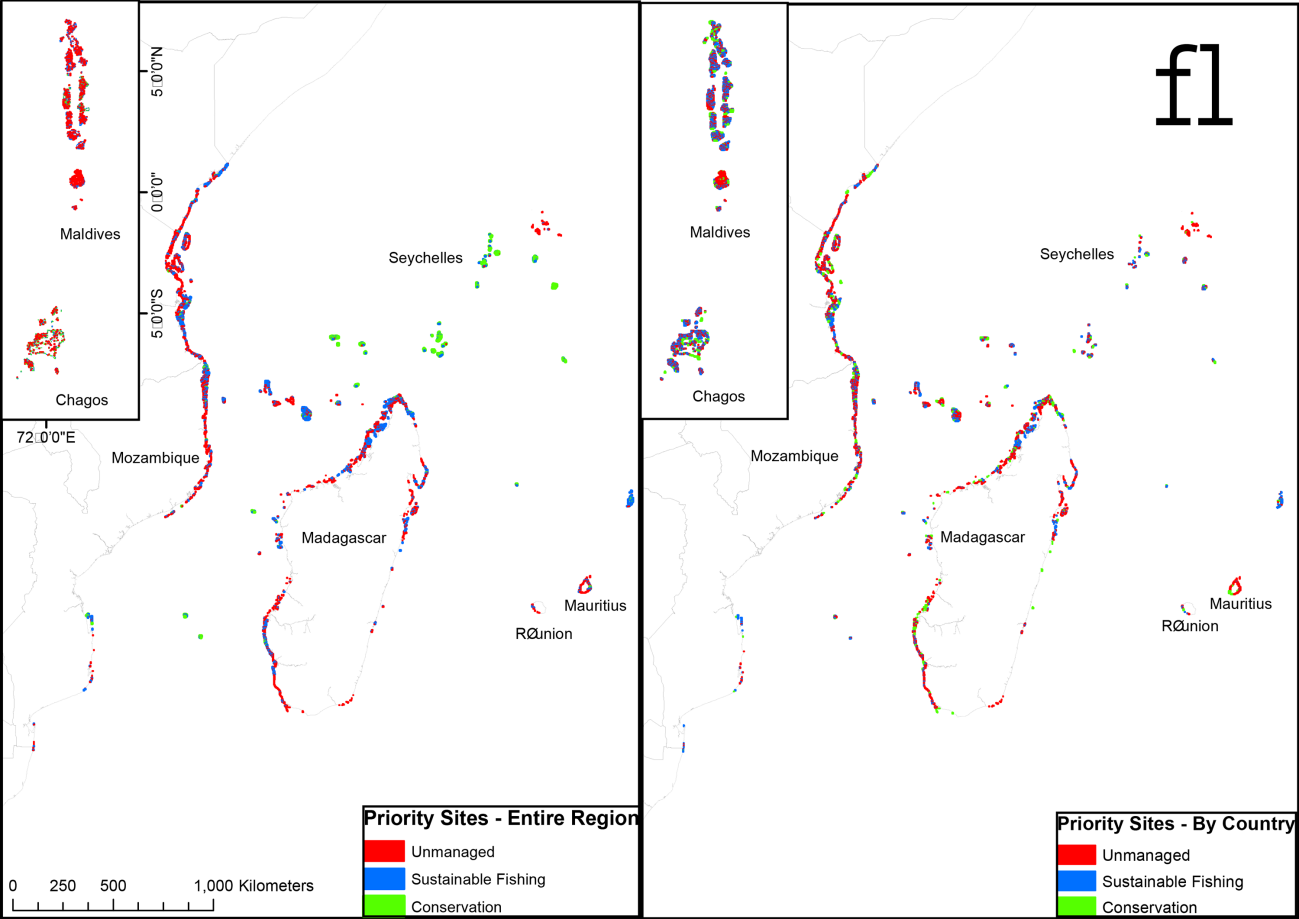
Western Indian Ocean maps of Marzone maximum probability priority selections for 50% sustainability, 20% conservation, and 30% unmanaged where to time to recovery was the cost and minimized if (a) countries collaborated to reach these goals, and (b) there was no collaboration between countries.

Applying the Marxan algorithms to minimize time to recovery and establishing the spatial goals of 20% of the reefs for conservation and 50% for sustainability, partitions these three target management categories differently depending on the by-country and entire-region coordination scenarios (Fig 6). The entire-region scenario has most of the conservation areas placed in the offshore island of the Maldives, Chagos, and Seychelles but also some areas in northern Kenya and Mozambique and a few locations scattered throughout, including northern Madagascar (Fig 6A). The by-country scenario places the conservation areas more broadly, as 20% conservation has to be established in each country (Fig 6B). This results in new sites added in southern Tanzania and its offshore islands. Also, much of northern Madagascar is prioritized for conservation and all countries have sites selected according to where the highest biomass is predicted.

The times to recovery for the three governance scenarios indicate the fastest recovery for collaboration, followed by the no-country collaboration, and finally by the biomass depletion status (Table 5). The whole-region values to reach the sustainable yield thresholds were 0.43±0.51, 0.76±0.92, and 3.91±1.34 years for the collaboration, no collaboration, and local management governance scenarios, respectively. Maximum diversity would require 0.66±0.77, 1.43±0.91, and 6.24±1.14 and conservation thresholds 3.27±2.14, 7.13±2.53, and 15.23±2.17 years for the three governance scenarios, respectively. Again, these values vary considerably by location, country, threshold, and scenario.

**Table 5:** Mean (±SD) time to recovery (in years) for sustainable yields (450 kg/ha), maximum diversity (600 kg/ha), and conservation (1150 kg/ha) thresholds for core closure priority areas. Marxan was used to select reefs for the regional collaboration, and no- collaboration scenarios, while the degradation prioritisation is described in the methods). NS indicates countries where Marxan/Degradation prioritisation did not select any conservation areas.

	Marxan—Regional Collaboration			Marxan—No Collaboration			Degradation Prioritization		
Country/Thresholds	450 kg/ha (SD)	600 kg/ha (SD)	1150 kg/ha (SD)	450 kg/ha (SD)	600 kg/ha (SD)	1150 kg/ha (SD)	450 kg/ha (SD)	600 kg/ha (SD)	1150 kg/ha (SD)
Bassas da India	0 (0)	0 (0)	0 (0)	0 (0)	0 (0)	0 (0)	NS	NS	NS
British Indian Ocean Territory	0 (0)	0 (0)	0.12 (0.54)	0 (0)	0 (0)	0.04 (0.29)	NS	NS	NS
Comoro Islands	NS	NS	NS	4.78 (0.7)	6.86 (0.49)	15.66 (0.84)	4.27 (1.08)	6.49 (0.8)	15.04 (1.47)
Glorioso Islands	0.53 (1.51)	0.78 (2.2)	1.78 (5.01)	1.29 (2.03)	1.94 (3.03)	4.44 (6.95)	4.47 (0.28)	6.58 (0.24)	15.04 (0.54)
Ile Europa	0 (0)	0 (0)	0 (0)	0 (0)	0 (0)	0 (0)	NS	NS	NS
Ile Tromelin	0 (0)	0 (0)	0 ()	0 (0)	0 (0)	0 (0)	NS	NS	NS
Juan de Nova Island	0 (0)	0 (0)	0 (0)	0 (0)	0 (0)	0 (0)	NS	NS	NS
Kenya	0.38 (0.91)	1.55 (2.42)	5.02 (6.02)	0.65 (0.96)	3.46 (1.77)	11.2 (2.08)	4.04 (2.7)	6.54 (2.15)	16.99 (5.51)
Madagascar	2.98 (2.62)	5.21 (2.66)	13.61 (4.23)	4.27 (1.83)	6.4 (1.8)	15.42 (3.5)	4.56 (1.48)	6.72 (1.29)	15.82 (2.79)
Maldives	0 (0)	0 (0)	0.11 (0.81)	0.06 (0.4)	0.47 (1.22)	5.11 (4.19)	1.04 (1.32)	3.66 (1.61)	11.59 (1.32)
Mauritius	1.02 (2.35)	1.34 (3.08)	3.12 (7.18)	4.22 (2.78)	5.56 (3.65)	12.94 (8.51)	6.07 (0.39)	7.98 (0.35)	18.66 (1.06)
Mayotte	0 (0)	0 (0)	0 (0)	0.62 (0.95)	4.23 (0.63)	11.83 (0.62)	2.07 (0.82)	5.03 (0.48)	12.69 (0.66)
Mozambique	2.86 (2.25)	4.25 (3.26)	9.76 (7.49)	4.84 (1.36)	6.88 (1.39)	16.03 (2.75)	5.12 (1.1)	7.16 (0.95)	16.62 (2.13)
Reunion	NS	NS	NS	0.29 (0.66)	2.25 (2.22)	8.73 (5.02)	3.39 (2.68)	5.8 (2.57)	15.26 (4.58)
Seychelles	0 (0)	0 (0)	0 (0)	0.26 (1.11)	0.37 (1.57)	0.85 (3.59)	4.55 (0.91)	6.69 (0.69)	15.39 (1.31)
South Africa	NS	NS	NS	0 (0)	0 (0)	5.12 (0)	NS	NS	NS
Tanzania	1.51 (1.09)	4.68 (0.72)	12.32 (0.79)	3.03 (1.79)	5.67 (1.24)	13.86 (2.06)	3.45 (1.96)	5.95 (1.46)	14.46 (2.48)
**Regional average**	**0.66 (0.77)**	**1.27 (0.8)**	**3.27 (2.14)**	**1.43 (0.91)**	**2.59 (1.01)**	**7.13 (2.53)**	**3.91 (1.34)**	**6.24 (1.14)**	**15.23 (2.17)**

Regional collaboration generally shortens countries time to recovery, but the differences can vary. For example, regional collaboration decreased recovery time by up to 9 years in countries such as Mozambique and Mauritius (Table 5). Some countries, such as Mauritius or the Comoros, have very low biomass overall, such that none of their reefs would be included in a regional collaboration while others, such as Seychelles, already have many of their reefs above thresholds and therefore require less time or costs to participate in the collaboration. Conversely, the biomass-depleted prioritization approach requires a long recovery period for Kenya and Mauritius but so do many of the countries with low biomass, most countries requiring 3 to 6 and 12 to 18 years to achieve the mean sustainable yield and conservation thresholds, respectively.

## Discussion

Conservation planners and managers are faced with different approaches to prioritizing marine conservation that can vary based on underlying philosophies and values of what is important to protect, for what reasons, by whom, and how best to promote effective human actions [40–42]. Typically, a common concern and the main use of systematic conservation planning is the efficient use of limited resources and trade offs required to protect representative threatened biodiversity [37, 43]. Political boundaries are also a concern, as conservation requires collective action and most operate at some political level ranging from local coastal communities, such as fish landing sites, sub-national divisions, nations, regional and global governance bodies [44, 45]. Proposed planning should stimulate human actions that have some measurable and predictable effect on ecosystems and human livelihoods. Consequently, technical planning needs to contribute to larger portfolio of decision-making activities, which should include factors not easily modeled in spatial plans but also by approaching planning with a variety of assumptions and associated scenarios.

Here, we present the spatial conservation prioritization outcomes of a variety of potentially common management approaches. The spatial prioritization plans deriving from these different philosophies can vary but overlap enough to form a basis for comparison and compromises [41, 43]. We emphasize the importance of developing a portfolio of approaches where hidden values and associated cultural decisions are included in the models. Because these values are hidden in model assumptions, they are often a source of conflict when technical solutions are presented in subsequent deliberative discussions [46, 47]. Many resource conflicts and failures to implement technical solutions occur when technical solutions have not fully appreciated access issues, or psychological and cultural values that produce difficult-to-quantify trade offs[48, 49].

A common management approach is to preferentially protect areas having the highest conservation potential at the minimum cost [50]. This is, for example, the approach being used by nations and some conservation organizations that prioritize remote and intact areas that can quickly reach conservation target areas, including the Chagos Archipelago in the Indian Ocean [51]. Large and remote protected areas support significantly different biotic communities from national closures, particularly the protection of apex predators and scraping herbivores that are often uncommon in the more typical national closures that are frequently developed for ecotourism purposes [9]. Standard prioritization has the advantages of efficiency and affordability but there are also hidden transaction and opportunity costs of negotiations, enforcement, and monitoring. Further, they can lack redundancy and a political balance of costs and responsibilities that may be critical for accommodating failures and political efforts to establish regional protected areas [52–54].

An example from this region is that the probability of closure failure is likely to be >35% as indicated by the ratio of low compliance to total closures reported in recent WIO regional surveys [6]. Failure probability will also vary in different social environments and, while low economic development is often associated with high biomass reefs, the capacity to protect it is likely to be limited without significant intervention [55, 56]. Similarly, very remote areas may lack stakeholder communities willing to maintain the costs of their protection. Designations can be motivated by the desire to achieve targets and be unrealistic about the efficacy and social justice issues created when remote protected areas with large expenditures are created [54]. Social injustice can be leveled at these cases, as scarce resources might be better spent on stakeholders that benefit from the establishment of protected areas.

Here, we see some specific regional issues likely to arise when the least costly international collaboration is considered as a means to reach conservation targets. In this case, some countries are exempt from responsibility or action either because they already have reached the stated goals, such as the Seychelles, or the time to recovery are too long to require efficient action, such as Mauritius, while a burden can be added to others with limited resources, such as Tanzania. Here, other considerations are needed; for example, Mauritius has the highest level of fish endemism in the region and so any consideration of endemism would prioritize the Mascarene Islands [57, 58]. Tanzania has a history of conflict and low compliance with large national protected areas and any extra burden may require involvement of alternative livelihoods [11, 59]. These examples can typify regional issues that are not easily solved or policed by a regional oversight body. Consequently, while this approach is useful and may be a good way to insure some wilderness areas are identified and protected, they may fail to reach the appropriate level of effective governance, agreement, and social justice associated with collective expenditures that are considerations required for spatial planning [50, 60]. Similar challenges have been encountered through the well studied coral triangle initiative, potentially providing useful lessons for regions such as the western Indian Ocean

The no-national coordination results are more realistic about the appropriate scale of governance for some types of protected areas. They produce responsibility for each nation and its stakeholders, but do so in a way that conservation goals can be rapidly reached. While the recovery time required in this scenario is greater than the regional goal, for Madagascar, the differences are small and never more than 1 year. Costs would likely be offset by the time spent coordinating on a regional agreement and monitoring system. For example, despite a few efforts to create cross boundary protected area over the past decade, none of these efforts have produced concrete actions [23]. Further, there is not a high level of economic and governance interaction within the region that often precedes and is associated with regional conservation action [22, 39]. Countries in this region vary in the amount of area they already have and have proposed for protected areas [61] and have variable local social support for protected areas [6].

The final biomass-depletion selection scenario focuses on restoring degraded ecosystems that should improve fisheries and ecosystem resilience when restored. Models suggest that fisheries closures are only effective at increasing fisheries yields when biomass is reduced below MMSY levels [62–65]. Consequently, the biomass depletion approach fits well with these objectives in selecting sites that have biomass below MMSY levels. Fish biomass, diversity, and ecosystem services are often closely linked in coral reefs and therefore this planning approach is expected to produce other social-ecological benefits [5, 38, 66]. This approach does require the greatest recovery time, especially if the goal is for closures to reach the conservation threshold. Further, small closures have limited capacity to restore apex predators and other feeding functional and life histories groups [6, 9]. Nevertheless, there is evidence that governments and communities in the region are embracing and expanding this management tool [21, 56]. The success rate and full social-ecological outcomes needs further investigation but preliminary evaluations are hopeful in finding ecological changes and social acceptability for small closure sizes [67, 68].

### Model limitations

The spatial models have a number of limitations that need to be considered when evaluating their usefulness. The data used to build the model are well replicated and collected over large areas but there is variation in the fish biomass predictions by human population, management, and recovery rates that limit the predictive ability. There are likely to be differences at any specific sites that are not accounted for in the model, including habitat and environmental conditions that will influence reef fish biomass and recovery rates and also cultural factors, such as adoption, enforcement, and compliance. Other studies suggest that there are, however, clear and predictable relationships between distance to markets, management, and fish biomass [69, 70]. Further, fish recovery rates are often found to occur at a 15 to 25 year rate [17, 71, 72] but there are also reports of slower and faster recovery [7, 73]. Given that the model was calibrated with data collected in this region, the chances for errors of extrapolation is reduced. Nevertheless, many ecological and social factors are not well understood let alone modeled accurately on the regional scale of this study. Therefore, any application of the spatial model to specific sites will have to consider these limits. Local variability requires applying the usual social and ecological considerations during the planning and implementation process [40, 74].

The cost used here is recovery time, which is proportional to lost fisheries production or biomass not captured and consumed by people. Estimates of fisheries production are variable but generally fall ~4–6 tons/km^2^/year but can reach more than 10 tons/km^2^/year [55, 75]. Consequently, a loss of one year of fishing can represent around 0.5 million tons of reef fish for the region. This creates challenges to feeding or creating alternative food sources in an already biomass dependent and depleted fishery [48, 66]. Full closure to all fishing is, however, an extreme case used to estimate costs and less severe restrictions, such as gear management would allow partial recovery while still providing food and income [76]. This would extend the recovery time but is expected to create less social resistance.

Spatial prioritization tools are for decision support not decision making, which requires human experience and the inclusion of more criterion than are typically modeled [50]. Our study demonstrates the use of understanding of baselines, carrying capacity and rates of biomass recovery and associated ecological factors to identify and plan priority areas. Yet, we also show how different assumptions and proposals can lead to very different spatial priorities and foresee potential conflicts. From our analysis and the current state of governance in the region, we suggest that a combination of the by-country prioritization and the biomass depletion selection criteria is most likely to be adopted. These approaches fit the regions need to protect intact ecosystems and biodiversity at the national level but also sustain biomass and support the production of local fisheries [48, 66].

The methods that we used have potential to be applicable globally. Human coastal population densities are estimated at 1.2 x 10^9^ or nearly three times higher the global average and 17% of them rely on fisheries as a primary source of nutrition [28]. Clearly, the human population, market, and management factors shown here and elsewhere are largely driving the depletion of biomass of reef ecosystems globally [7, 69, 77]. Yet, one of the key outcomes of coral reef research in this region is that thresholds of fish biomass are critical for maintaining the ecological state and services [5, 6]. It is suggested that maintaining ecological states above the sustainability thresholds will provide greater potential to adapt to disturbances that will increase with global climate change. An important step in providing this adaptation potential is to develop spatial plans and priority-areas for conservation action that utilize these empirically derived thresholds.

## Supporting Information

S1 Fig Classification of reefs by their management categories used for estimating biomass. (JPG)

S2 Fig High resolution map of the western Indian Ocean for (a) modeled biomass based on the empirical relationship established in Fig 1., (b) the estimated time to recover biomass to a mean estimated sustainability level (450 kg/ha). Recovery rates are based on studies of biomass recovery in fully protected fisheries closures studied over a 20-year period (McClanahan et al. 2007).
(EPS)

S3 Fig High resolution map of the Western Indian Ocean for (a) the estimated time to recover biomass to a mean estimated sustainability level (600 kg/ha), and (b) the estimated time to recover biomass to the estimated conservation target of 1150 kg/ha. Recovery rates are based on studies of biomass recovery in fully protected fisheries closures studied over a 20-year period (McClanahan et al. 2007).
(EPS)

S4 Fig High resolution map derived from algorithm identifying and prioritizing the most depleted fish biomass for small closures and adjacent spillover reefs until all reefs with biomass <450 kg/ha are classified. (EPS)

S5 Fig High resolution maps of *Marzone* maximum probability priority selections for 50% sustainability, 20% conservation, and 30% unmanaged where to time to recovery was the cost and minimized if (a) countries collaborated to reach these goals, and (b) there was no collaboration between countries.
(EPS)

S1 Table A model selection table showing the 95% confidence set of models. (DOCX)

S2 Table Summary statistics of the nls models. (XLSX)
