# Correction: Modeling Reef Fish Biomass, Recovery Potential, and Management Priorities in the Western Indian Ocean

**DOI:** 10.1371/journal.pone.0156920

**Published:** 2016-06-01

**Authors:** Timothy R. McClanahan, Joseph M. Maina, Nicholas A. J. Graham, Kendall R. Jones

Due to an image conversion error, Figs 3–6 erroneously show the letters “fl” in their upper right-hand corners, rather than a compass symbol. Please view the corrected Figs 3–6 here.

**Fig 3:**
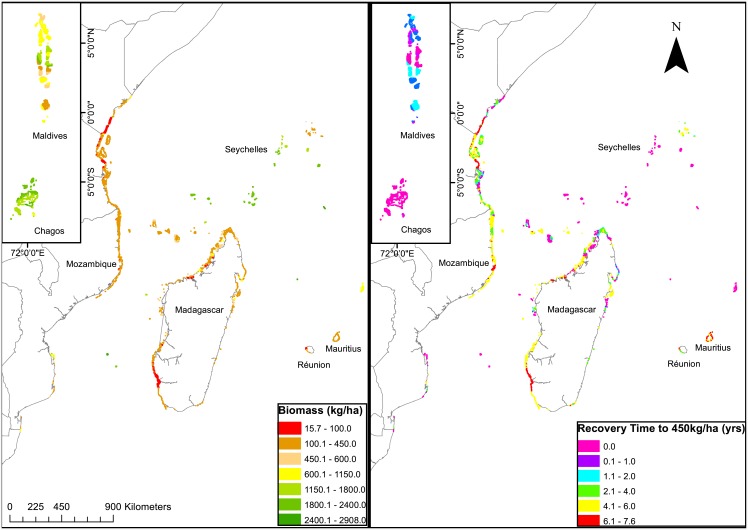
Map of the western Indian Ocean for (a) modeled biomass based on the empirical relationship established in Fig 1, and (b) the estimated time to recover biomass to a mean estimated sustainability level (450 kg/ha).

**Fig 4:**
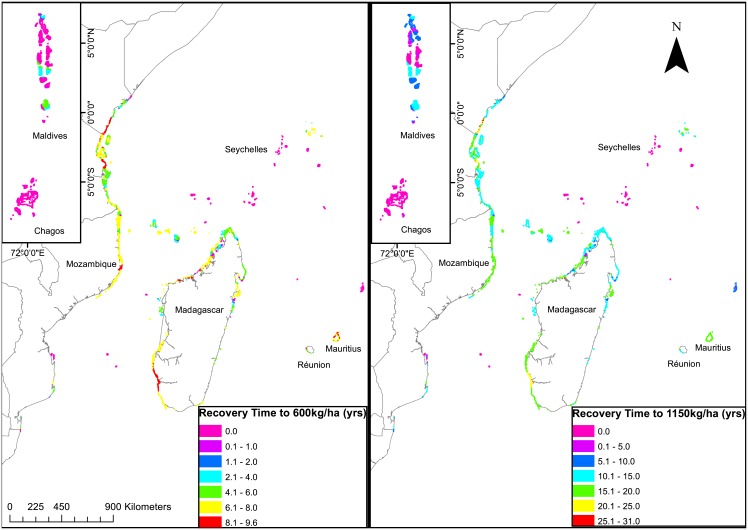
Map of (a) the estimated time to recover biomass to a mean estimated sustainability level (600 kg/ha), and (b) the estimated conservation target of 1150 kg/ha in fully protected fisheries closures studied over a 20-year period (McClanahan et al. 2007).

**Fig 5:**
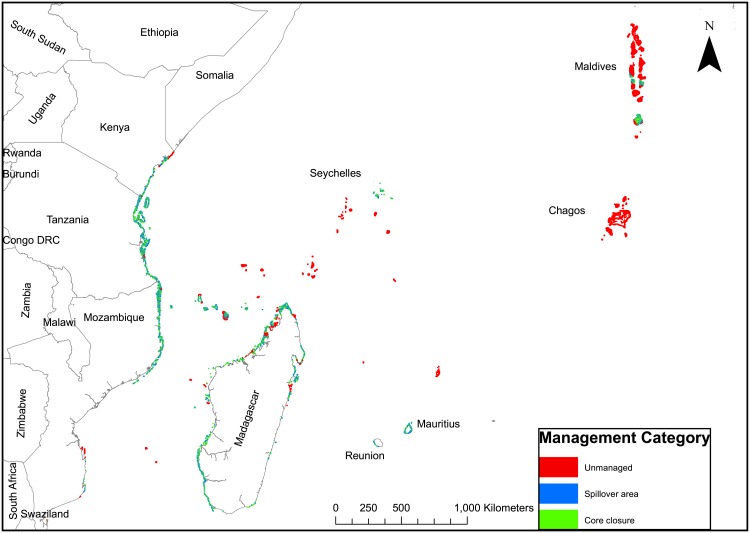
Map derived from algorithm identifying and prioritizing the most depleted fish biomass for small closures and adjacent spillover reefs until all reefs with biomass <450 kg/ha are classified.

**Fig 6:**
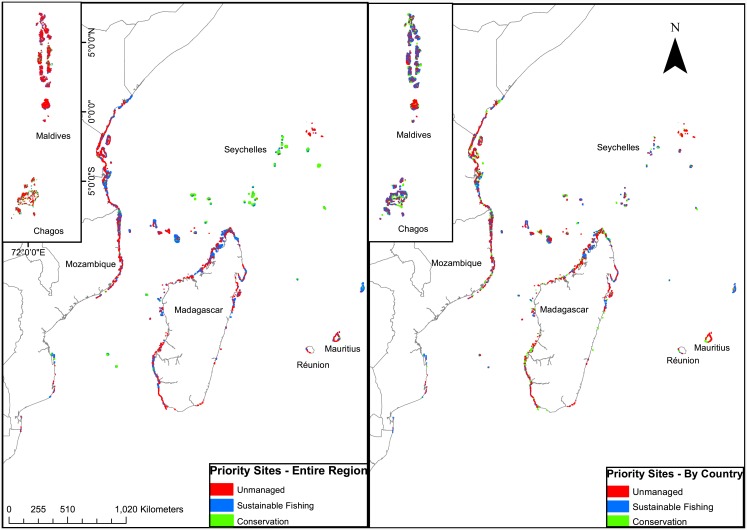
Western Indian Ocean maps of Marzone maximum probability priority selections for 50% sustainability, 20% conservation, and 30% unmanaged where to time to recovery was the cost and minimized if (a) countries collaborated to reach these goals, and (b) there was no collaboration between countries.
