# Supplementary figures and images for: Modeling Reef Fish Biomass, Recovery Potential, and Management Priorities in the Western Indian Ocean
Source: PLoS One. 2016 May 5;11(5):e0154585. doi: 10.1371/journal.pone.0154585 (PMC4858301; doi:10.1371/journal.pone.0154585)

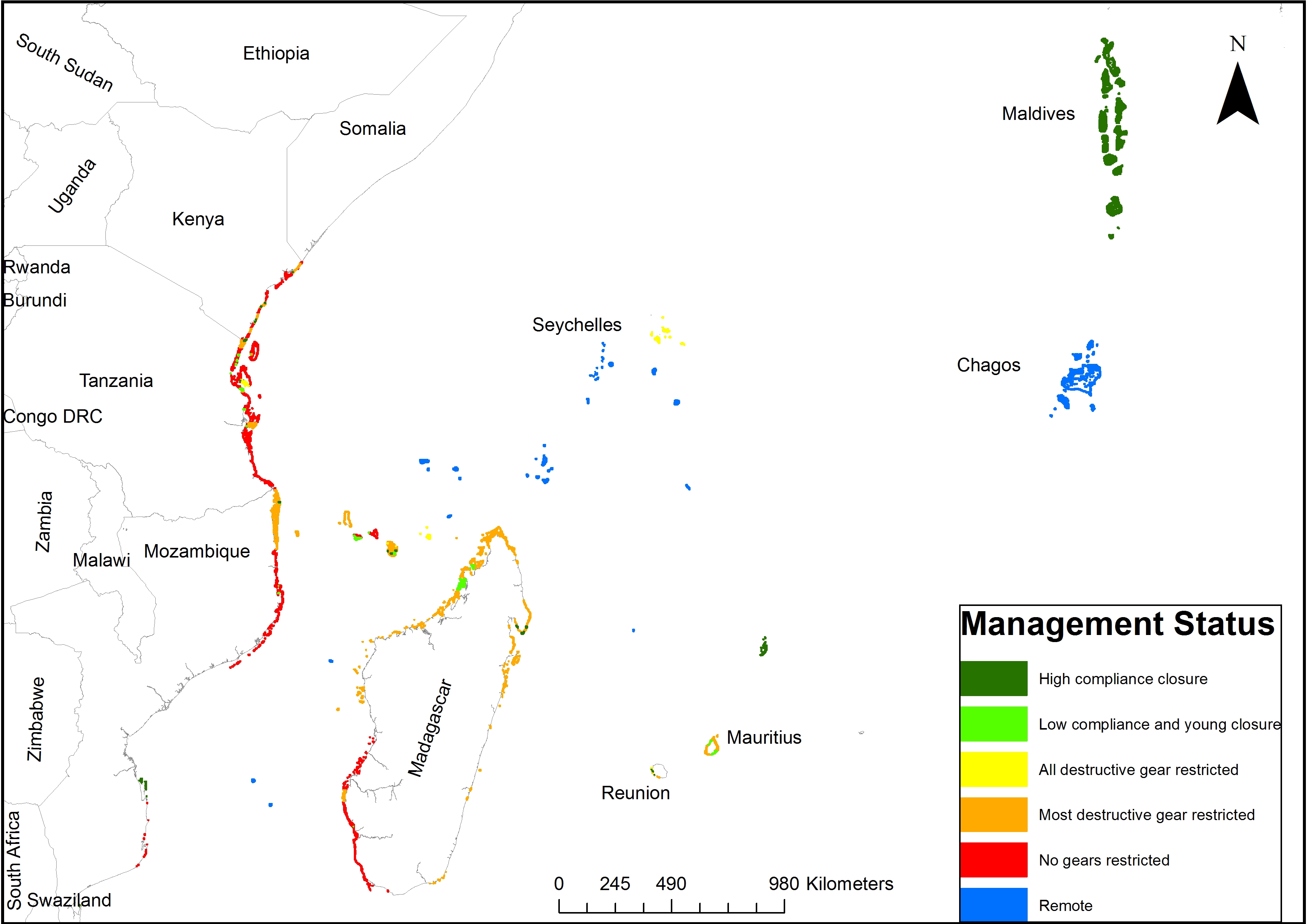

Supplement: S1 Fig — (JPG) [file pone.0154585.s001.jpg]
