# Supplementary material for: Modeling Reef Fish Biomass, Recovery Potential, and Management Priorities in the Western Indian Ocean
Source: PLoS One. 2016 May 5;11(5):e0154585. doi: 10.1371/journal.pone.0154585 (PMC4858301; doi:10.1371/journal.pone.0154585)
Supplement: S1 Table — (DOCX) [file pone.0154585.s006.docx]

| Model# | X.Intercept. | Fishing proxy | Management | Distance*fishing | Population | Max SST | R^2^ | Adjusted  R^2^ | df | logLik | AICc | delta | weight |
| --- | --- | --- | --- | --- | --- | --- | --- | --- | --- | --- | --- | --- | --- |
| 1 | 5.5 |  | + | + |  | + | 0.6 | 0.7 | 14.0 | -665.0 | 1358.9 | 0.0 | 0.5 |
| 2 | 5.3 | + | + | + |  | + | 0.6 | 0.7 | 15.0 | -664.2 | 1359.2 | 0.3 | 0.4 |
| 3 | 5.5 |  | + | + | + | + | 0.6 | 0.7 | 16.0 | -664.9 | 1362.7 | 3.8 | 0.1 |
| 4 | 5.3 | + | + | + | + | + | 0.6 | 0.7 | 17.0 | -664.0 | 1363.2 | 4.3 | 0.1 |

Table S7. The 95% confidence set of models showing the corresponding goodness of fit and model inference statistics.
